# Investigation of non-uniform airflow signal oscillation during high frequency chest compression

**DOI:** 10.1186/1475-925X-4-34

**Published:** 2005-05-19

**Authors:** Kiwon Sohn, Warren J Warwick, Yong W Lee, Jongwon Lee, James E Holte

**Affiliations:** 1 Department of Electrical and Computer Engineering, University of Minnesota, Minneapolis, MN, USA; 2 Department of Pediatrics, University of Minnesota, Minneapolis, MN, USA

## Abstract

**Background:**

High frequency chest compression (HFCC) is a useful and popular therapy for clearing bronchial airways of excessive or thicker mucus. Our observation of respiratory airflow of a subject during use of HFCC showed the airflow oscillation by HFCC was strongly influenced by the nonlinearity of the respiratory system. We used a computational model-based approach to analyse the respiratory airflow during use of HFCC.

**Methods:**

The computational model, which is based on previous physiological studies and represented by an electrical circuit analogue, was used for simulation of *in vivo *protocol that shows the nonlinearity of the respiratory system. Besides, airflow was measured during use of HFCC. We compared the simulation results to either the measured data or the previous research, to understand and explain the observations.

**Results and discussion:**

We could observe two important phenomena during respiration pertaining to the airflow signal oscillation generated by HFCC. The amplitudes of HFCC airflow signals varied depending on spontaneous airflow signals. We used the simulation results to investigate how the nonlinearity of airway resistance, lung capacitance, and inertance of air characterized the respiratory airflow. The simulation results indicated that lung capacitance or the inertance of air is also not a factor in the non-uniformity of HFCC airflow signals. Although not perfect, our circuit analogue model allows us to effectively simulate the nonlinear characteristics of the respiratory system.

**Conclusion:**

We found that the amplitudes of HFCC airflow signals behave as a function of spontaneous airflow signals. This is due to the nonlinearity of the respiratory system, particularly variations in airway resistance.

## Background

High Frequency Chest Compression (HFCC) [6,9,19,29] is a useful and popular therapy for clearing bronchial airways of excessive or thick mucus since it does not require patients to do any directed efforts for respiration while therapy is given, unlike other airway clearance techniques such as active cycle of breathing or autogenic drainage [17]. A HFCC machine pumps air into an inflatable jacket worn by patient. By means of a surrogate piston, sine waveform compression pulses with frequencies ranging from ~5 Hz to ~21 Hz are supplied to the thorax of a patient through the jacket. These pulses squeeze and vibrate the patient's thorax at prescribed frequencies. These actions help in the evacuation of mucus through changing the rheological property of the mucus and airflow oscillation. King et al. [13], Krumpe et al. [15], and Tomkiewicz at al. [28], showed that HFCC pulses decrease the viscosity of mucus and helps evacuation. A more important consequence is the respiratory airflow oscillated during use of HFCC, which results from variation of intrapleural pressure. Lapin [17] and Warwick [30] pointed out that airflow is the most important factor for mucus transport since airflow produces the shear stresses for evacuation of mucus.

Although several models have been developed to simulate respiration by other researchers and their models successfully worked for their own purposes, these models are not appropriate for HFCC simulation because these models were either linearized [7,8] or because they assumed that respiration was driven by a mechanical ventilator at the mouth [5,20]. The model presented in this paper provides reliable simulation results on fast change of intrapleural pressure (*P*_*pl*_) altered by HFCC because this model is described with nonlinear equations and *P*_*pl *_is selected as the driving force of respiration. The nonlinear characteristics of the respiratory system are not easily noticed during quiet tidal breathing, but the airflow signal oscillation measured at the mouth during use of HFCC is a strong indicator of the nonlinear characteristics of the respiratory system.

In this study, we simulated the respiratory system with a computational model that carefully reflected its nonlinear characteristics. The model is an electrical circuit analogue, in which nonlinear resistors (R's), capacitors (C's), and inductors (L's) represent airway resistance, lung capacitance, and inertance of air, respectively. Just like in a living organ, the driving force of this model is *P*_*pl *_which is a superposition of HFCC pulses on the spontaneous breathing effort. We compared the simulation data to the *in vivo *data to demonstrate and understand the characteristics of airflow signal oscillation.

## Methods

### Conceptual model of the respiratory system

Our modelling and simulation for reproducing airflow signals required simplification of airway structure in the lung. The geometrical and dimensional structures of the airways were proposed by several researchers, among which Horsefield et al. [10] and Weibel [31] dissected and measured the human lungs and airways, and more recently, and Tawhai et al. [11] and Kitaoka et al. [14] proposed algorithmic approaches for reconstructing the branching structure. We employed Weibel's morphometry of the lung, which provides the geometries and the dimensions based on the symmetric dichotomous structure of airway branching when the lung volume is assumed to be 75% of the total lung capacity (*TLC*). According to his morphometry, the trachea is defined as airway generation 0 and it is separated into two geometrically identical daughter branches. Each daughter is repeatedly branched up to 22 times, thus the lung is considered to have 24 (0–23) airway generations and the number of airway branches total ~1.7 × 10^7^. In each airway generation, there are 2^*z *^(z is a generation number) identical branches whose lengths and radii are provided and the dimensions of airway branches in each generation differ from generation to generation. He also suggested that airway generations 0–16 comprise the conducting zone, whereas airway generations 17 – 23 make up the respiratory zone in which gases are exchanged.

**Figure 1:**
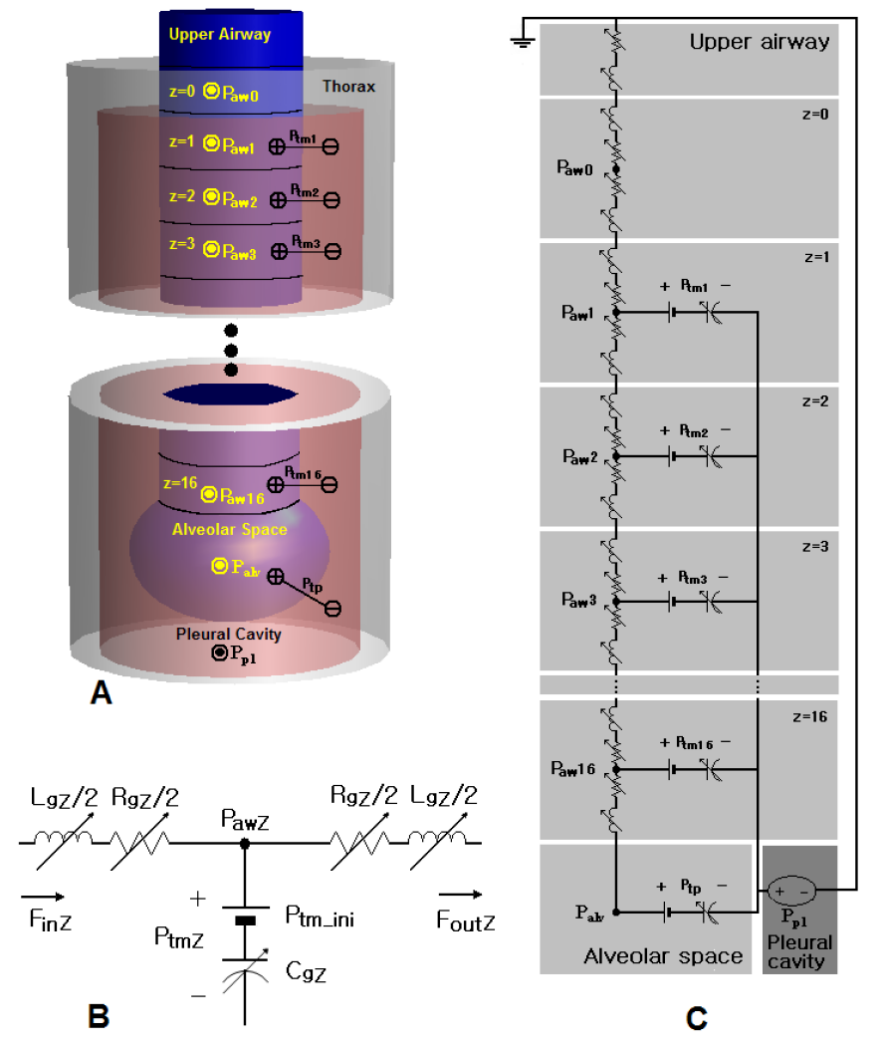
(a) The conceptual model based on Weibel's morphometry of the lung [31]. The lung and airways are comprised of five regions, namely, the alveolar space, the conducting airway zone, the pleural cavity, the thorax outside the lung, and outside the thorax. (b) RCL T-network that represents a region (airway generation) in the conducting airway zone. (c) Electrical circuit analogue converted from (a). For acronyms in the figure, see list of abbreviations.

Based on Weibel's morphometry of the lung, we simplified the geometry of the airways. For airway generation *Z *in conducting zone, a bundle of 2^*z *^identical airway branches are considered as the big tube whose cross sectional area equals to 2^*z *^times the cross sectional area of a single airway branch. The tubes for each airway generation are represented by RCL T-networks shown in fig. 1 (b), and evaluation of the R's, C's, and L's in the RCL T-network are explained in equations (4) – (6). Meanwhile, the respiratory zone is considered as a big lump, the alveolar space, since alveolar ducts and sacs are scattered throughout the respiratory zone [8]. Although the upper airway is not presented in Weibel's morphometry, it is one of the chief sites for airway resistance.

Our conceptual model as a summary of the simplification, 'the upper airway + 17 conducting airway generations + the alveolar space', is demonstrated in fig. 1 (a), and circuit analogue converted from fig. 1 (a) is shown in fig. 1 (c).

### Dimensions of airway branches

Since the dimensions of every single airway branch in the lung vary during respiration, it is crucial to track them to extract proper values of R's, C's, and L's. The radii of the airway branches in airway generation *Z *are determined by transmural pressure (*P*_*tmZ*_), pressure difference between the pleural cavity and inside airway generation *Z*. In order to determine *P*_*tmZ*_, we need to rely on the study about transpulmonary pressure (*P*_*tp*_), pressure difference between the pleural cavity and alveolar space. According to Salizar et al. [26], *P*_*tp *_is a function of lung volume (*LV*) and *TLC*. With given *LV*, *P*_*tp *_is obtained by,

*P*_*tp *_= -*log *(1 - *LV */ *TLC*) × 7.22.     (1)

If *LV *is functional residual capacity (*FRC*) and no airflow exists in the airways, the lung is at rest. In this situation, air pressure at any site is the same and atmospheric (zero), therefore *P*_*tmZ *_and *P*_*tp *_are the same. Our simulation begins with assuming that the lung was at rest. The equation of Lambert et al., or Lambert's tube law [16], is a function of *P*_*tmZ*_, and gives the ratio of the cross sectional area (*A*_*Z*_) to the maximum cross sectional area of an airway branch (*A*_*maxZ*_) in airway generation *Z*. That is,

*A*_*Z *_/ *A*_max *Z *_= 1.0 - (1.0 - *α*_0_)(1.0 - *P*_*tmZ *_/ *P*_0_)^-*N *^    (2)

where *α*_0_, *α*_0_' and *N *are constants for each airway generation given from Lambert's tube law, and *P*_0 _= (*α*_0_-1)*N*/*α*_0_'. As mentioned earlier, the dimensions of the airway branches provided by Weibel's morphometry are based on 75 % of *TLC*. Let *A*_*Z75 *_be Weibel's cross sectional areas in airway generation *Z*. By using



*A*_*maxZ *_for each airway generation can be found. Since *A*_*maxZ *_does not change whether or not the lung is at rest, the equation is valid to find out *A*_*Z *_during respiration.

The length of an airway branch is assumed to vary in away that;

*l*_*z *_/ *l*_*FRC *_= *r*_*z *_/ *r*_*FRC *_    (3)

where *l*_*z *_and *r*_*z *_are the length and the radius of an airway branch in airway generation *Z*, respectively. *l*_*FRC *_and *r*_*FRC *_are the length and the radius on *FRC*, respectively.

### Equivalent circuit elements modelling

The RCL T-network consists of two resistors (*R*_*gZ*_), two inductors (*L*_*gZ*_), a capacitor (*C*_*gZ*_), and a DC voltage source (*P*_*tm_ini*_). *R*_*gZ *_and *L*_*gZ *_are the airway resistance and the inertance of air in the entire airway generation *Z*, respectively. *C*_*gZ*_, the airway capacitance of the entire airway generation *Z*, represents inflation and deflation of the non-rigid airway wall during respiration. *P*_*tm_ini *_is the initial value of *P*_*tmZ*_. This initial pressure counterbalances *P*_*pl*_, which is negative when the lung is at rest.

The values of the circuit elements in the RCL T-network of airway generation *Z *can be obtained by totalling the 2^*z *^airway branches. Therefore, *R*_*gZ *_is given by



Similarly, *L*_*g*_*z *is given by



and *C*_*gZ *_is

*C*_*gZ *_= *C*_*sZ *_× *N*_*z *_[ml/cmH_2_O],     (6)

where *R*_*sZ*_, *L*_*sZ*_, and *C*_*sZ *_indicate the values of a single airway branch in airway generation *Z*. *N*_*z *_is 2^*z*^, the number of airway branches in generation *Z*.

*R*_*sZ *_depends on the classic Poiseuille equation and the *Zeta *correction factor proposed by Pedley et al. [21]. The *Zeta *correction factor is given by



and



*R*_*e *_is Reynolds number , *V *is air velocity, *ρ *is the density of air, and *η *is the viscosity of air.

Because electric current flows at the speed of light in an electrical circuit, it is necessary to compensate for the incomparably slower behaviour of airflow in the airways by placing inductors. Inertance of air in a single airway branch (*L*_*sZ*_) depends on the dimension of the airway branch [5] and it is given by



**Figure 2:**
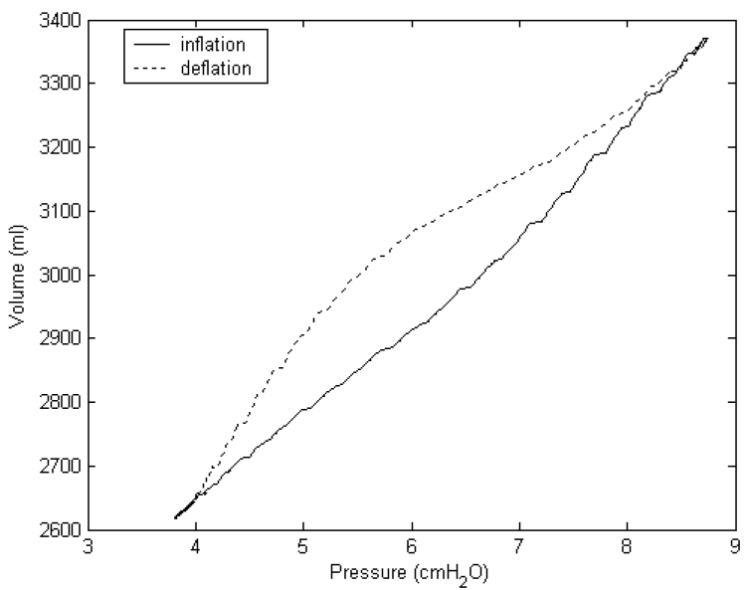
*P*_*tp*_-volume curves of the lung model during use of HFCC. Although the initial *LV *(*FRC*) is 2700 ml, *LV *during use of HFCC is smaller. The curves became jagged due to HFCC pulses.

To compute the values for *C*_*sZ *_in equation (2), relation between *A*_*Z *_and *P*_*tmZ *_in airway generation *Z*, is used again. That is,





The alveolar space is represented by a capacitor in the circuit analogue. Alveolar capacitance (*C*_*as*_) is the ratio of the alveolar volume (Δ*AV*) to Δ*P*_*tp*_. Lung volume (*LV*) consists of airway volume and alveolar volume, and airway volume is negligible compared to alveolar volume. Therefore,



which implies that  represents *C*_*as*_. It can be obtained by equation (1); however, the equation did not consider the hysteresis of *P*_*tp*_-volume curves that is caused by several proposed reasons [1]. To overcome this, we defined *C*_*as *_as  *k (*k*<0), and *k *was continuously changed over the time course of the simulation. The values of *k *were empirically determined to achieve the acceptable shape of the hysteresis, which is shown in fig. 2.

A resistor and an inductor characterize the upper airway, from the nasal/oral cavity to larynx. The equations for the resistance of the upper airway (*R*_*ua*_) that Jackson *et al. *[12] validated are described below:



where *F*_*ua *_is the airflow rate in the upper airway. As Marchal et al. [18] estimated, the value of the inductor that represents inertance of air is 0.00003 [cmH_2_O·s^2^/ml].

### Numerical methods for nonlinear circuit analogue

**Figure 3:**
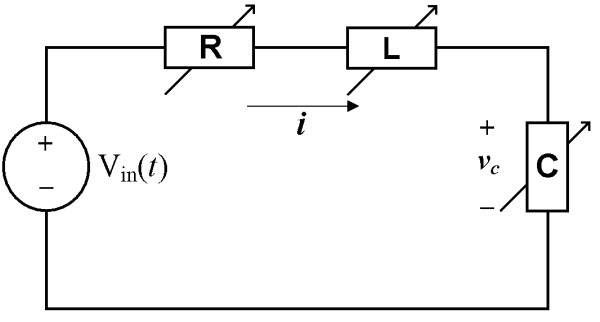
A nonlinear second-order system in the form of an electrical circuit. *i *is electrical current, and *v*_*C *_is voltage difference between each end of *C*. The value of the resistor (R) in this figure is dependant on *i *as well as *v*_*C *_whereas the capacitor (C) and the inductor (L) are dependent only on *v*_*C*_. V_in_(*t*) is a voltage source for this simple circuit system. Nonlinear circuit elements in this circuit imply the nonlinearity of fig. 1 (c).



Although the number of elements is manageable and the structure of circuit is fairly simple, analysis of the circuit analogue in fig. 1 (c) is not trivial since the values of all the energy-storing (C's and L's) elements as well as resistive elements (R's) change at every sequence of the simulation time-step. To exemplify a general idea of numerical methods for the nonlinear circuit analogue, analysis of a simple nonlinear second-order system in fig. 3 was demonstrated. The system of fig. 3 is described by an equation:

where *v*_*C *_is the voltage difference between each end of the capacitor. Note that *R*(•) is a function of *i *and *v*_*C*_, and *L*(•) and C(•) are functions of *v*_*C *_just like in fig. 1 (c). Using backward Euler approximation [22], equation (14) is converted to a difference equation:



where Δt is time difference between sequence [n] and [n-1]. Equation (15) is the same as



By the definition of backward Euler approximation, the current of the circuit . Then equation (16) can be restated as an equation;



In equation (17), the right hand side consists of all known values, and the left hand side is a function of *v*_*C*_[n]. Suppose that *v*_*C*_[n] is *x*, equation (17) can be expressed as

*f*(*x*) = c,     (18)

where c is a constant. Equation (18) can be easily solved using an iteration method [24]. To do this computation, MATLAB (Mathworks, Natick, MA) codes were written.

### Protocols for measurement airflow signals

**Figure 4:**
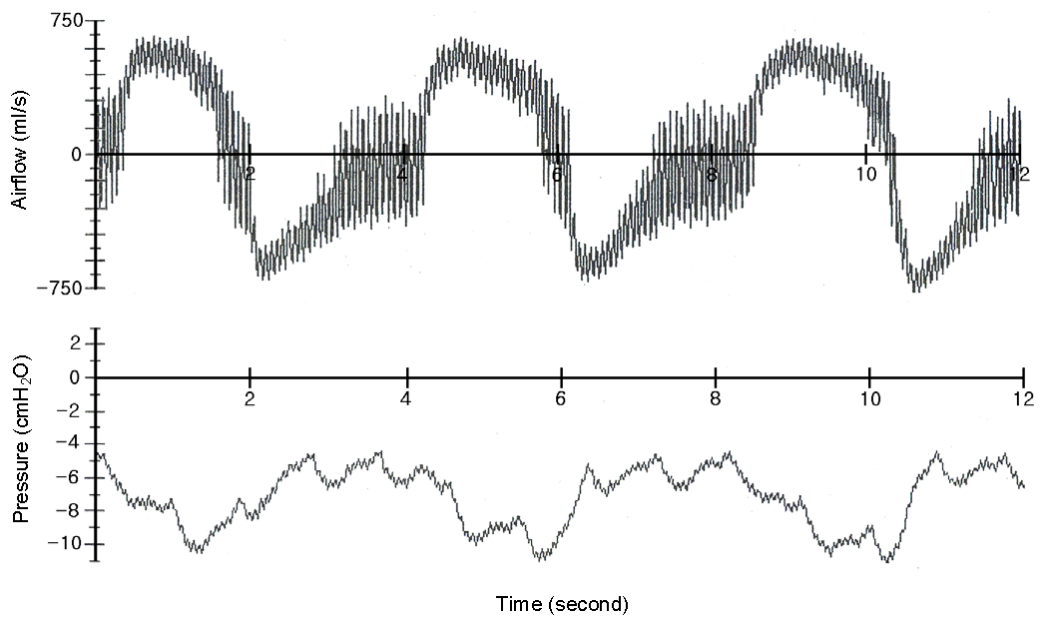
(a) The airflow and (b) the oesophageal pressure measured by Fink et al. [6] (modified with the permission), which shows typical respiratory airflow during use of HFCC. The positive numbers of airflow rate represent inspiration and the negative numbers represent expiratory airflow. Oesophageal pressure reflects the intrapleural pressure (*P*_*pl*_) that is altered by HFCC pulses (tiny peaks).

In this study, *The Vest*™ (Advanced Respiratory, St.Paul, MN, USA; now named Hill-Rom Co.,Inc.), which delivers sine waveform compression pulses, was used for application of HFCC to a subject for measuring and recording the airflow signals at the mouth. The general usage of HFCC device was early described in the review of Hansen et al. [9] and the typical respiratory airflow during use of HFCC is shown in fig. 4.

The subject sat upright on a chair for measuring and recording the airflow signals at the mouth with an in-house built electronic spirometer. The subject worn a nose clip and breathed through a mouthpiece. After the HFCC device is properly set up, the compression pulses were applied and then the subject made several slow and large, but not to *TLC*, breaths. During the breaths the subject hold his glottis open until data collection was completed. This protocol was followed for the low (5 Hz), high (21 Hz), and medium frequencies (15 Hz) of HFCC pulses. Before each frequency recording of airflow signals, the subject rested for one minute. To ensure that the subject had adapted to HFCC pulses and had reached a steady state, only the last ten seconds of the one- minute breathing were recorded and analyzed for our study.

## Results

For the simulation, the parameters of the lung were determined to represent a normal healthy lung. The *TLC*, *FRC*, and *RV *of the model lung are 6000 ml, 2700 ml, and 1000 ml, respectively. The ambient atmospheric pressure is assumed to be zero, and the air density and viscosity are 0.00113 g/cm^3 ^and 0.00019 g/cm·s, respectively. It was assumed that there would be no airflow in the airways and that initially the *LV *would be the same as the *FRC*. Change of *P*_*pl *_is the primary driving force of respiration and is initially -4.32 cmH_2_O, which is the counterbalance to initial *P*_*tp *_given by equation (1).

**Figure 5:**
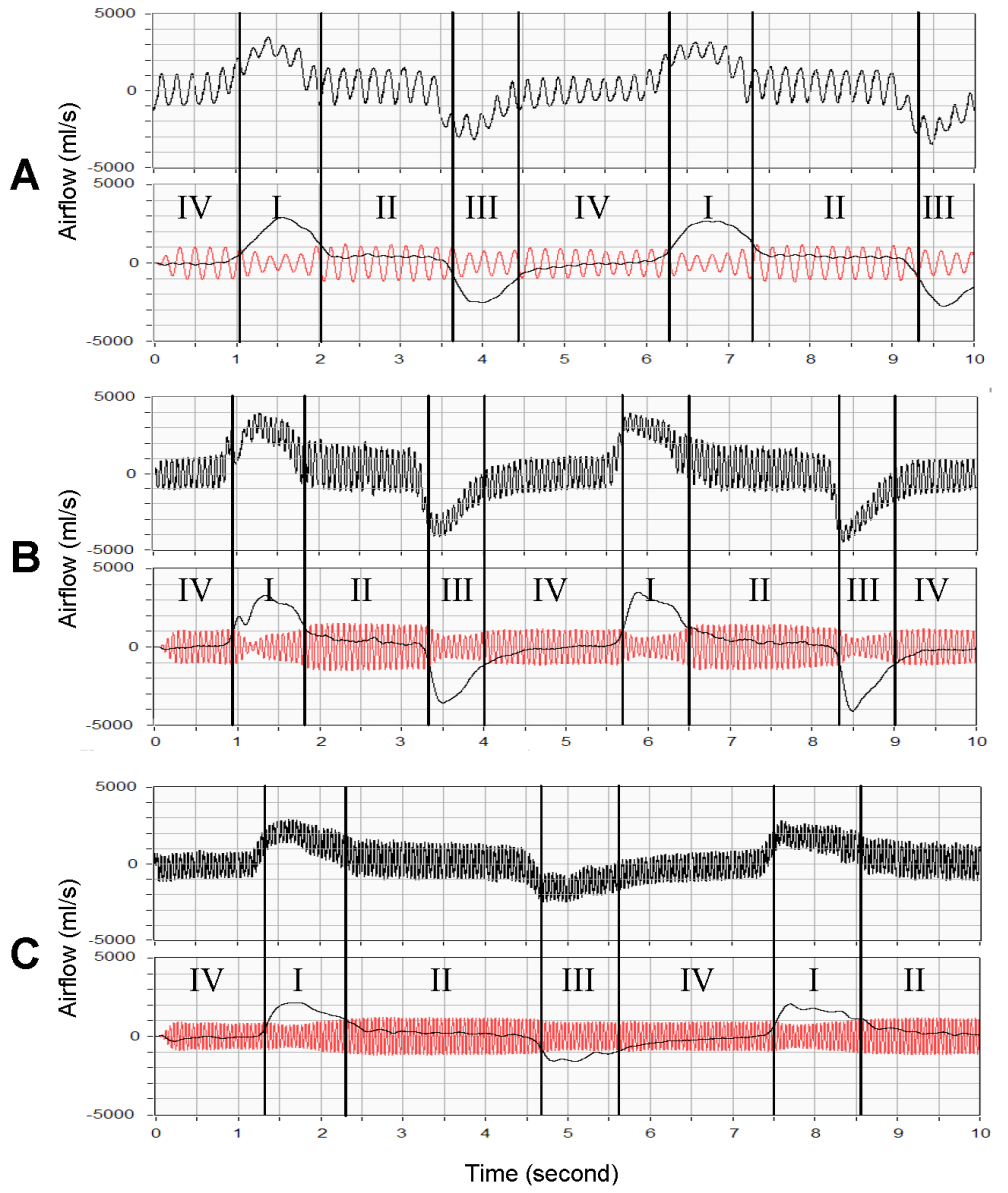
The airflow signals measured at the mouth of the subject, the high-pass filtered (HFCC airflow signal) and the low-pass filtered (spontaneous airflow signal) curves. The subject was using HFCC with (a) 5 Hz, (b) 15 Hz, and (c) 21 Hz. Regardless of the frequencies, larger spontaneous airflow signals result in smaller HFCC airflow signals. Since it is difficult to breathe hard during 21 Hz, spontaneous airflow signals in (c) are smaller than in (a) and (b). Phase I is the portion of the inspiration phase when spontaneous airflow signals are greater than the amplitudes of HFCC airflow signals. During phase II, spontaneous airflow signals stay within the amplitudes of HFCC airflow signals. In phase III, expiration begins, and the amplitudes of airflow oscillation decrease to about the amplitudes during phase I. Spontaneous airflow signals get greater to the negative direction than HFCC airflow signals. Finally phase IV, the resting period before inspiration, begins, and the amplitudes of HFCC airflow signals again become greater than spontaneous airflow signals. To emphasize the difference of HFCC airflow signals, phase II were consciously prolonged and they are longer than phase II in fig. 6.

**Figure 6:**
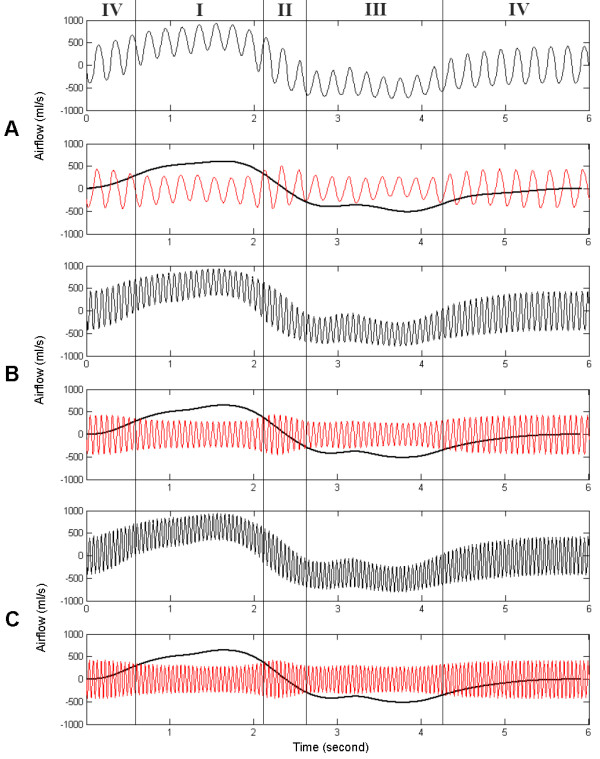
Simulated respiration, spontaneous airflow signals and HFCC airflow signals. The frequencies of HFCC were set (a) 5 Hz, (b) 15 Hz, and (c) 21 Hz. Definitions for Phases I, II, III, and IV in this figure are the same as those in the text and fig. 5.

Fig. 4 is the airflow the oesophageal pressure measured by Fink et al. [6], which shows typical respiratory airflow during use of HFCC. Fig. 5 shows the subject's airflow signals during ten seconds at the three different frequencies. In this figure, each airflow signal is also viewed as the low-pass filtered and the high-pass filtered curves. The high-pass filtered curve indicates the fast airflow signal oscillation generated by HFCC pulses (HFCC airflow signal) whereas the low-pass filtered curve is the airflow during the spontaneous breathing effort (spontaneous airflow signal) of the subject. We divided one cycle of the respiration into four phases. Phase I is the portion of the inspiration phase when the spontaneous airflow signal is greater than the amplitudes of HFCC airflow signal. In this phase *LV *increases. During phase II, the spontaneous airflow signal stays within the amplitudes of the HFCC airflow signal. Phase II is the pause before expiration begins. The amplitudes of HFCC airflow signals change considerably as the phase moves from I to II. To emphasize the difference of HFCC airflow signals, phase II were consciously prolonged and they are longer than phase II in fig. 6. In phase III, passive or active expiration begins and the amplitudes of HFCC airflow signals decrease to about phase I amplitudes. The low-pass curve gets greater to the negative direction curve than the high-pass curve. The next and last phase is phase IV. Phase IV is the resting period before inspiration, begins. During this phase the amplitudes of HFCC airflow signals again become greater than the spontaneous airflow signal. To reproduce similar airflow signals, a cycle of respiration from phase IV to phase IV was simulated using our computational model. The simulation results with the three frequencies are shown in fig. 6.

From the measured values and the simulation of airflow signals at the mouth, we could observe two important phenomena during respiration pertaining to the airflow signal oscillation generated by HFCC. First, the amplitudes of HFCC airflow signals in phases I and III were smaller than those in phases II and IV. Second, the amplitudes in phases I and III became even smaller as the spontaneous airflow signal became greater.

**Figure 7:**
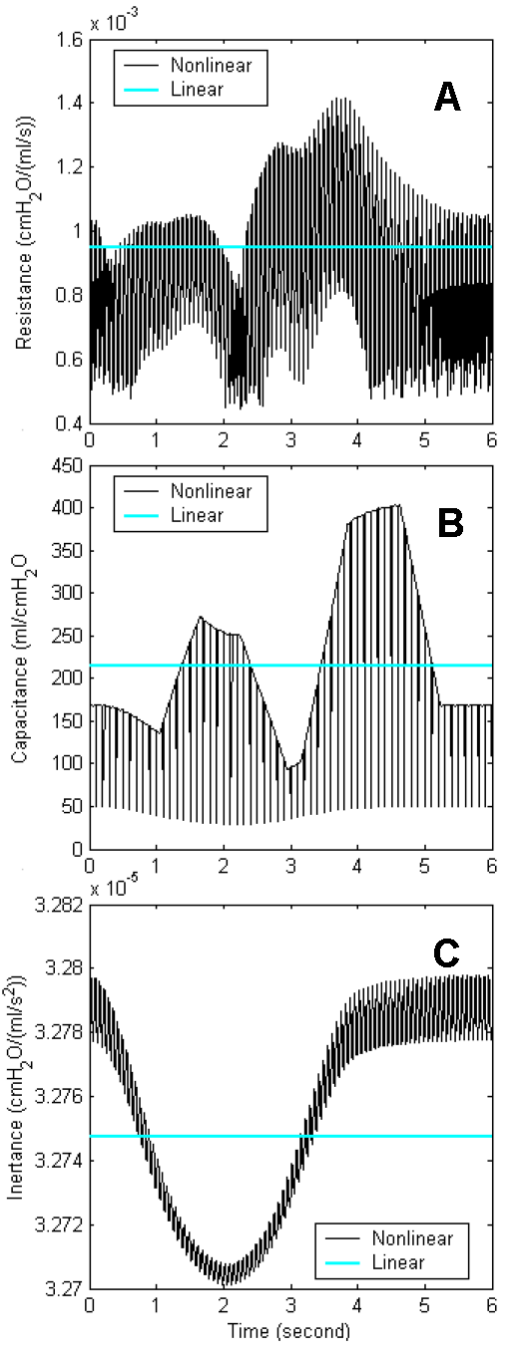
(a) Airway resistance, (b) lung capacitance, and (c) inertance of air during the simulation used for fig. 8. The linear (fixed) values are the mean values of each nonlinear (varying) value.

**Figure 8:**
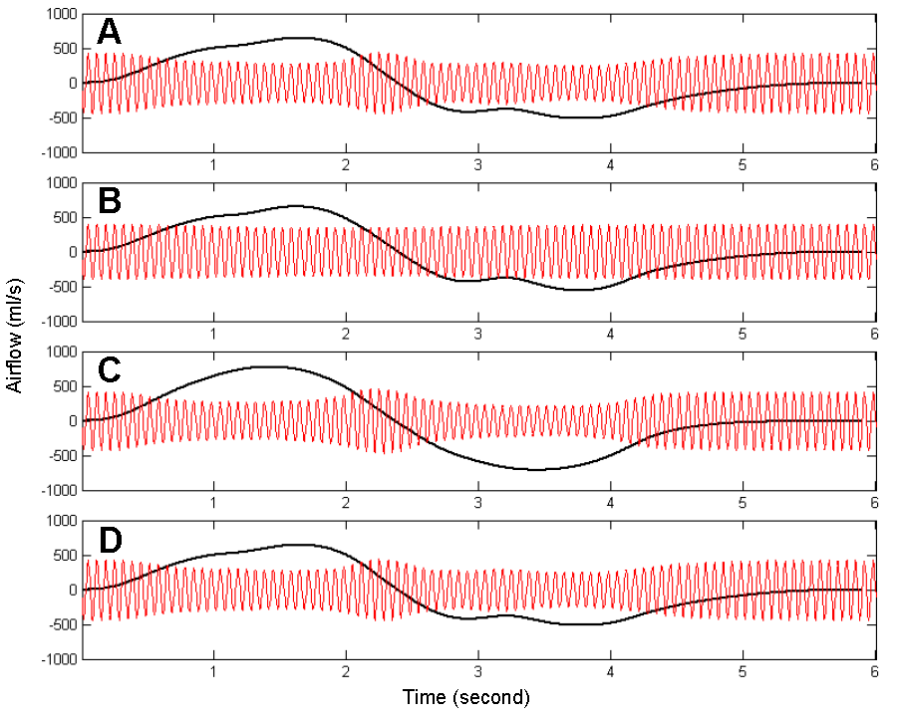
Simulated spontaneous airflow signals and HFCC airflow signals, (a) when the values of airway resistance, lung capacitance, and inertance of air are nonlinear (varying), (b) when the value of airway resistance is linear (fixed), (c) when the value of lung capacitance is linear (fixed), and (d) when the value of inertance is linear (fixed). The frequencies of each plot are 15 Hz, so (a) is the same as fig. 6 (b). In (b), the amplitudes of HFCC airflow signals almost do not vary.

**Figure 9:**
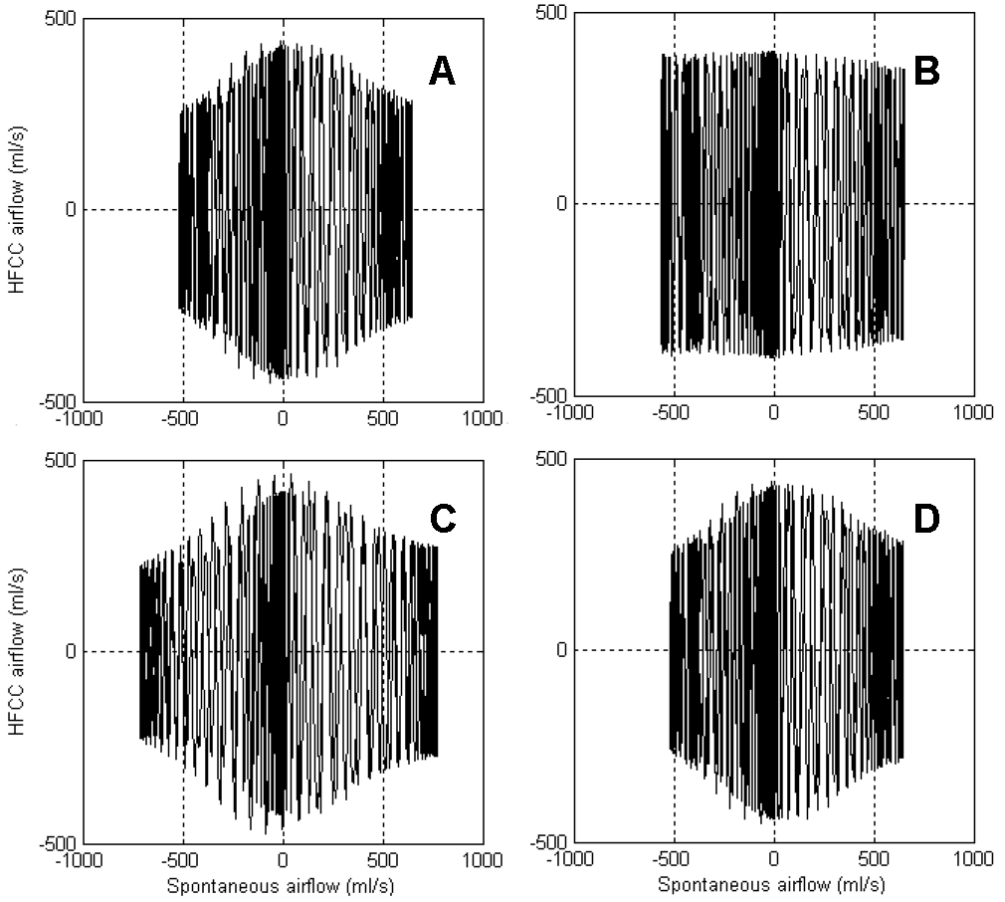
HFCC airflow signals are demonstrated as a function of spontaneous airflow signals based on the same simulation data shown in fig. 8. Nonlinear airway resistance (a, c, and d) produces the non-uniform amplitudes of HFCC airflow signals.

We used the simulation results to investigate how the nonlinearity of airway resistance, lung capacitance, and inertance of air characterized the respiratory airflow. The simulation was repeated after setting one of the three properties as a linear constant value. Fig. 7 compares the linear values with the nonlinear values of the three properties. The linear values are means of the nonlinear values during the simulation. Fig. 8 presents the simulation results of the lung model under the imaginary assumptions. Fig. 8 (a) is normal respiratory airflow at 15 Hz, and fig. 6 (b) and fig. 8 (a) are from the same simulation data. Fig. 8 (b) demonstrates the predicted airflow signals at the mouth with linear airway resistance, which indicates the amplitudes of HFCC airflow signals do not vary significantly. Fig. 8 (c) is the airflow when lung capacitance is set to a linear value. Just like fig. 8 (a), HFCC airflow signals are the largest when spontaneous airflow signals are close to zero. Therefore, it can be presumed that the nonliearity of lung capacitance does not play a role in the non-uniformity of HFCC airflow signals. And neither is inertance of air. Fig. 8 (d), which shows the simulation data with linear inertance of air, is almost identical to fig. 8 (a). This indicates that the inertance of air is also not a factor in the non-uniformity of HFCC airflow signals. Fig. 9 shows the HFCC airflow signals as a function of spontaneous airflow signals based on the same simulation data shown in fig. 8. Fig. 8 (a), (c), and 8 (d) indicate that larger spontaneous airflow signals result in smaller oscillations of HFCC airflow signals whereas HFCC airflow signals do not seem to be related to spontaneous airflow signals in fig. 8 (b).

## Discussion

The number of the airway branches is estimated to be about 17 million [31], and the dimensions of each airway branch vary while the lung is being inflated and deflated during respiration. To reduce the computational burden, models of the respiratory system are often simplified by ignoring the nonlinear natures of the respiratory system [7,8]. Linearization entails considerable flexibility in numerical analysis since linear circuits are computationally much cheaper than nonlinear circuits, either on a time-domain or a frequency-domain basis. However, even during the slowest breathing, airway resistance, lung capacitance, and inertance of air change due to the variation of airway dimensions as well as turbulence. In particular, increase of the airway resistance due to turbulence is so drastic that inaccurate evaluation of airway resistance may result in a misleading simulation result. Our simulation results demonstrated that such errors are more likely when HFCC intervention is applied. Evidence for this was that the result from a linear model was significantly different from that from a nonlinear model (fig. 8 and 9). As parts of the respiratory system, lung capacitance and inertance of air are also nonlinear although it was observed that their nonlinearity did not cause the non-uniform amplitudes of HFCC airflow signals. However, this does not imply that the nonlinear characteristics of lung capacitance and inertance of air have no role in the simulation of the respiratory system. Dimensional changes of the airway branches in the lung are responsible for the nonlinearity of lung capacitance and inertance of air. When dimensions of the airways are involved for simulation and prediction, the nonlinearity is very important for accurate results. For example, in the study of Sohn et al. [27], our model is used for estimation of air velocity, which is airflow rate ÷ cross sectional area. Since the cross sectional areas of airway branches do not vary linearly, lung capacitance and inertance of air also should not be linear in order to avoid discrepancy.

Ideally, studies about airflow in the airways would be best resolved by CFD (Computational Fluid Dynamics), however, using a CFD approach in this study presents several problems that cannot be overcome by modern technology. First, the turbulence mechanism is not completely known [23]. Even during the slowest breathing manoeuvre, turbulence exists in the proximal airways, and ignoring turbulence would not give true simulation results. Another obstacle is that the whole lung cannot be taken into consideration even with the latest supercomputing power. A CFD approach to simulate dynamics of airflow interacting in a huge number of airway branches in the lung requires extremely massive computation. There is no way to deal with it, if any, its computation time would be incredibly long. It should be also pointed out that CFD techniques for airflow in non-rigid wall tubes are not yet mature. Although not perfect, our circuit analogue model allows us to effectively simulate the nonlinear characteristics of the respiratory system.

It is well known that respiratory system impedance consists of airway impedance and chest wall (tissue) impedance [2-4]. Since the driving force of airflow in our model is the intrapleural pressure altered by HFCC pulses transferred from the body surface, our model does not necessarily incorporate chest wall (tissue) impedance. However, it is not clear how effectively the chest wall transfers HFCC pulses to the pleural cavity. Other factors should also be considered for impedance between the jacket of HFCC and the pleural cavity – such as clothes, posture, and tightness of the jacket. Currently, based on observations of Milla et al. [19], we assume that HFCC pulses on the body surface are transferred to the pleural cavity without any distortion of pulsation waveforms although some attenuation may exist.

The HFCC device was initially developed only for cystic fibrosis patients who normally have healthy lungs [29]; consequently we modelled a healthy lung as the first step of the research. As this medical treatment becomes widely applicable to other lung diseases, we are planning to develop models that can be used to simulate HFCC on various lung diseases in the future.

## Conclusion

In this study, the airflow signals measured at the mouth during use of HFCC are viewed as a composite of two causes: the spontaneous breathing effort and HFCC pulses. However, since the respiratory system is nonlinear, airflow signals at the mouth are not a mere superposition of the effects from these two causes. In laboratory measurements, the amplitudes of the airflow signal oscillation varied considerably despite the uniformity of the HFCC pulses. After confirming that the simulation results matched up with the observations, we analyzed the simulation data to explain the observed inconsistency in the HFCC airflow signal amplitudes. We found that the amplitudes of HFCC airflow signals behave as a function of spontaneous airflow signals. This is due to the nonlinearity of the respiratory system, particularly variations in airway resistance.

The findings in this paper may not be immediately applicable for HFCC therapy, but they do lead to ways to better prescribe HFCC therapy. Most importantly, the usefulness of our computational simulation and the model-based approach as a tool to understand clinical observations of HFCC was well demonstrated in this paper.

## List of abbreviations

HFCC: High Frequency Chest Compression

*FRC*: Functional Residual Capacity

*TLC*: Total Lung Capacity

*RV*: Residual Volume

*LV*: Lung volume

*AV*: Alveolar volume

R: resistor

C: capacitor

L: inductor

*A*_*Z*_: Airway cross sectional area in airway generation *Z*

*A*_*maxZ*_: Maximum Airway cross sectional area in airway generation *Z*

*A*_*Z75*_: Airway cross sectional area in airway generation *Z *when *LV *is 75% of *TLC*

*C*_*as*_: Alveolar capacitance

*C*_*gz*_: Total airway capacitance in airway generation *Z*

*C*_*sZ*_: Single airway capacitance in airway generation *Z*

*F*_*inZ*_: Incoming airflow in airway generation *Z*

*F*_*outZ*_: Outgoing airflow in airway generation *Z*

*F*_*ua*_: Airflow in the upper airway

*L*_*gz*_: Total inductance (inertance of air) in airway generation *Z*

*L*_*sZ*_: Single inductance (inertance of air) in airway generation *Z*

*l*_*Z*_: The length of an airway branch in airway generation *Z*

*N*_*Z*_: Number of airway branches in airway generation *Z*

*P*_*alv*_: Alveolar pressure

*P*_*awZ*_: Airway pressure in airway generation *Z*

*P*_*pl*_: Pleural pressure or intrapleural pressure

*P*_*tm_ini*_: The initial value of transmural pressure

*P*_*tmZ*_: Transmural pressure in airway generation *Z*

*P*_*tp*_: Transpulmonary pressure

*P*_*tp_ini*_: The initial value of transmural pressure

*R*_*gz*_: Total airway resistance in airway generation *Z*

*R*_*sZ*_: Single airway resistance in airway generation *Z*

*r*_*z*_: The radius of an airway branch in airway generation *Z*

## Authors' contributions

KS designed all the necessary computational and experimental procedures, analysed data to reach the conclusion, and also prepared the texts and figures in the manuscript. WJW conceived this study, helped organizing the manuscript, and advised all aspects of physiology and medicine for this study. YWL and JL helped measuring the respiratory airflow. JEH advised all aspects of technology and engineering for this study.
